# Fatness and fitness: exposing the logic of evolutionary explanations for obesity

**DOI:** 10.1098/rspb.2015.2443

**Published:** 2016-01-13

**Authors:** Andrew D. Higginson, John M. McNamara, Alasdair I. Houston

**Affiliations:** 1 Centre for Research in Animal Behaviour, College of Life and Environmental Sciences, University of Exeter, Exeter EX4 4QG, UK; 2 School of Biological Sciences, Life Sciences Building, University of Bristol, 24 Tyndall Avenue, Bristol BS8 1TQ, UK; 3 School of Mathematics, University of Bristol, University Walk, Bristol BS8 1TW, UK

**Keywords:** obesity, thrifty genotype, thrifty phenotype, intake regulation, energy balance

## Abstract

To explore the logic of evolutionary explanations of obesity we modelled food consumption in an animal that minimizes mortality (starvation plus predation) by switching between activities that differ in energy gain and predation. We show that if switching does not incur extra predation risk, the animal should have a single threshold level of reserves above which it performs the safe activity and below which it performs the dangerous activity. The value of the threshold is determined by the environmental conditions, implying that animals should have variable ‘set points’. Selection pressure to prevent energy stores exceeding the optimal level is usually weak, suggesting that immediate rewards might easily overcome the controls against becoming overweight. The risk of starvation can have a strong influence on the strategy even when starvation is extremely uncommon, so the incidence of mortality during famine in human history may be unimportant for explanations for obesity. If there is an extra risk of switching between activities, the animal should have two distinct thresholds: one to initiate weight gain and one to initiate weight loss. Contrary to the dual intervention point model, these thresholds will be inter-dependent, such that altering the predation risk alters the location of both thresholds; a result that undermines the evolutionary basis of the drifty genes hypothesis. Our work implies that understanding the causes of obesity can benefit from a better understanding of how evolution shapes the mechanisms that control body weight.

## 1. Introduction

The mechanisms that control food intake have been the subject of much study in humans and other animals [1–5]. One important reason for this focus is the need to understand the causes of obesity in humans, a leading medical issue in many societies [2,6,7]. Research effort focuses on mechanisms of food seeking, meal duration, and feelings of hunger, and has suggested directions for interventions to help people lose weight [8]. However, such approaches have limited success in helping people manage their weight, as individual body weight tends to be resistant to change [8,9]. Reasons for such resistance have been sought by trying to understand the evolutionary pressures that lead to a phenotype that maintains adiposity in the face of environmental challenge [10]; i.e. by attempting to infer how natural selection in ancestral environments has resulted in human feeding strategies that now promote persistent obesity [11].

An important role of fat storage is to meet energetic needs when food intake is insufficient [5,12]. Such a shortfall may occur during the famines that have occurred throughout human history [13]. The thrifty genotype hypothesis [14] and the thrifty phenotype hypothesis [15] propose that animals, including humans, have energy storage strategies that enable them to survive such periods when food is scarce or not available. That is, people are genetically predisposed (thrifty genotype) or induced by early experience (thrifty phenotype) to eat excess food in times of plenty, so that they have sufficient stores for times of need. Following this strategy in the modern Western environment, which has been likened to a ‘continuous feast’ [16,17], can lead to obesity.

However, it is clear that individuals do not gain weight indefinitely, but tend to defend a defined level of energy reserves [18]. Such observations led to the set-point model of the control of adiposity [19], which was bolstered by the discovery of leptin, a hormone that appears to provide negative feedback from adiposity to eating behaviour [20]. The set-point model compares intake control to a system like a thermostat: if energy reserves are below a given threshold, the animal eats; if above that threshold, the animal does not eat. Because the change in body mass can be determined by the energy-balance equation of energy intake minus energy expenditure, it is possible to lose fat either by decreasing intake or increasing expenditure [21]. The set-point model is supported by the ineffectiveness, in the long term, of attempts to alter body weight through changes in energy intake and/or expenditure, because it suggests that the system will make compensatory adjustments to either intake or expenditure [10].

There is much evidence that is not compatible with the set-point model of control [17,22]. The critical phenomenon that any theory must address is the fact that in societies where many are obese, there is usually a majority that are not. While it is accepted that cognitive self-control will cause variation in body mass [17], much effort has been directed at finding non-cognitive explanations. The dual intervention point model [22] is an attempt at a model that is consistent with all the evidence. Under this view, humans have ‘evolved a regulatory system (i.e. a lipostatic system) that promotes fat storage to avoid starvation but also prevents excessive fat storage to avoid predation’ [23, p. 2098S]. In this model, the animal has two thresholds. The lower threshold prevents reserves from getting so low that the animal is in danger of starving. The upper threshold prevents reserves from getting so high that the animal is in danger of predation due to reduced manoeuvrability. Between the two thresholds the animal does not actively regulate reserves. Speakman [22] argues that the two thresholds are set independently and that the development of fire and society made humans safe from predators. This release from predation pressure allowed genetic drift in the location of the upper threshold, whereas the lower threshold has not moved, resulting in a wide distribution of human body mass. Progress in understanding obesity has frequently followed from research on the adaptive control of body fat [4,11,24]. In this article, we use novel results from models of optimal behaviour to expose the logic of models of the control of body adiposity.

## 2. The model

We constructed a computational, numerical model of a generic animal attempting to survive in an environment with a stochastic food supply. We model survival over an indefinite number of discrete time steps and find the decisions to forage or rest that minimize the rate of mortality. Decisions are assumed to be influenced by the animal's state. We characterize the state of the animal as its amount of energy reserves *x*, with some upper limit to what can be stored, *s*, such that 0 ≤ *x* ≤ *s*. Each time step the animal uses some energy to meet its metabolic needs. If its energy reserves drop to zero, the animal dies of starvation (cf. [12,25,26], see [27] for a review). At any given time, the animal is carrying out activity *A*, which is either *Low* (*L*) or *High* (*H*). These activities differ in the probability of being attacked by a predator, *D*_A_, and of finding a food item, *R*_A_, with *D*_L_ ≤ *D*_H_ and *R*_L_ ≤ *R*_H_. Finding food is stochastic such that the animal can be unlucky and have to rely on reserves to stay alive. We assume that food items vary in their energetic content, with mean energetic content *r* and variability *σ* (i.e. they contain the following units of energy: *r* − *σ*, *r*, *r* + *σ*). While we do not explicitly model famines, it is possible to obtain no food for long periods of foraging. We assume that the cost (predation risk) and rewards (probability of finding food) are positively associated, so that the animal always faces a trade-off between increasing the risk of starvation and increasing the risk predation. The animal manages its energetic reserves in order to minimize its total mortality rate.

We analyse the behaviour of the animal in two scenarios: (i) a forage/rest model where the animal can find food and be attacked by a predator only during *High* activity (*D*_L_ *=* 0, *D*_H_ *>* 0, *R*_L_ *=* 0, *R*_H_ *>* 0); i.e. activity *L* is resting in a safe refuge and activity *H* is foraging. We assume that the probability of predation in the refuge is zero; while this is unlikely to be true in reality, having a non-zero probability would not qualitatively change our results provided it was lower than the probability of predation during foraging; (ii) a two-location model where the animal can both eat and be attacked by predators during both activities (*D*_L_ *>* 0, *D*_H_ *>* 0, *R*_L_ *>* 0, *R*_H_ *>* 0). We parametrize the latter model such that when *x* = 0.5 *s*, activity *L* offers a small net loss of energy and a low predation risk, while activity *H* offers a small net gain of energy and a higher predation risk. This captures the idea that the animal faces a choice between losing weight but being relatively safe or taking increased risks in order to gain weight.

In each time step, the animal makes a decision whether to carry on with the current activity or switch to the other activity. Switching always takes one time step, and so is always costly in terms of the energy used in a time step, but switching may also carry a greater risk of predation than foraging. Switching is necessary if the animal is to start or stop foraging, or start to exploit a different food source. The predation risk may differ between switching, resting, and foraging, but in all cases increases with reserves, due to increased vulnerability because of reduced mobility [25,28–30], all else being equal (i.e. lean mass and so power-generating potential is unchanged). If the animal continues with the current activity the risk of mortality from predation to the animal is given by2.1
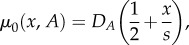
where *D_A_* is the probability of predator attack while doing activity *A.* If the animal decides to switch the mortality from predation is2.2
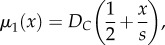
where *D_C_* is the probability of predator attack when switching. The term *x*/*s* implies that predation is mass dependent; specifically, an animal with maximum reserves (*x* = *s*) is thrice as likely to be killed by a predator as an animal with no reserves (*x* = 0). In order to model the situation where switching does not incur an added risk of predation we assume *μ*_1_ = 0 for all *x*. We assume that the metabolic energy cost paid by the animal *m*(*x*) also increases with energy reserves *x*, as in models of human weight management [31] according to2.3
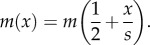
Note that we assume energy use does not depend on the activity.

We use standard state-dependent modelling methods [32,33] to find the optimal behaviour of the animal and then Markov chains to study the behaviour of these strategies (see the electronic supplementary material, appendix A for details). Electronic supplementary material, table A1 summarizes the parameters in the model and their default values.

## 3. Results

Generally, we find that when switching between the two activities carries no risk of predation, there is a single threshold level of reserves at which the animal should change from foraging to resting or *vice versa.* That is, below a certain level of reserves the animal should attempt to increase fat reserves; otherwise, it should allow fat reserves to decrease (cf. [12,25]). When switching involves a risk of predation, the strategy comprises two critical thresholds of reserves *c*_L_ and *c*_H_ (*c*_L_ ≤ *c*_H_). As the extra predation risk associated with switching increases, these thresholds get further apart. When reserves are in between the two thresholds (*c*_H_ ≥ *x* ≥ *c*_L_) the animal continues what it was doing in the last time step. When reserves hit the upper threshold *c*_H_ the animal switches from *H* to *L* and remains in *L* even if reserves go above *c*_H_. When the energy reserves reach the lower threshold *c*_L_ the animal switches from *L* to *H* and remains in *H* if reserves go below *c*_L_. Below, we describe the insights for understanding obesity that these models provide.

### (a) Forage/rest model

**Figure 1.**
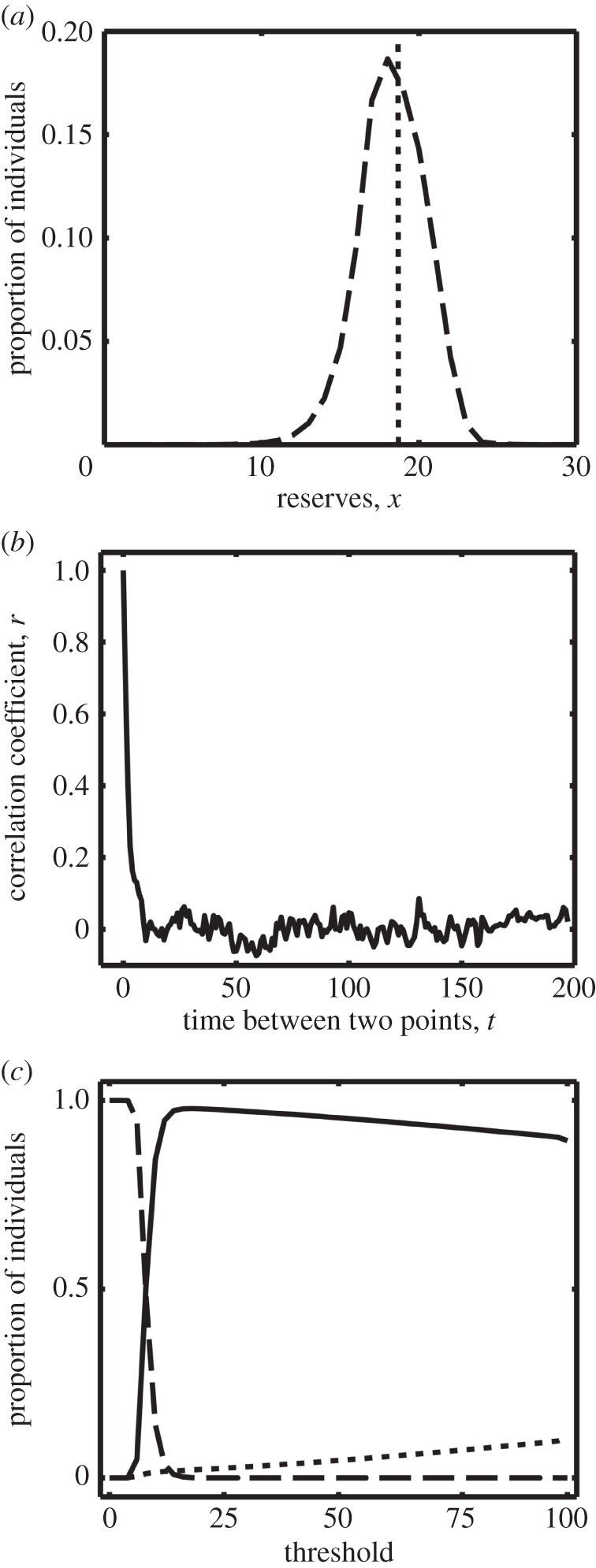
(*a*) Distribution of reserves (dashed line) for a large population of individuals following the optimal strategy of foraging or resting. The optimal strategy is to forage when *x* ≤ 19 (dotted line) and rest otherwise. Reserves can exceed the threshold by the maximum amount of energy contained in food (*r* + *σ*), and can drop below the threshold due to the stochastic nature of finding food. (*b*) Correlation coefficient between reserves at *p* and reserves at *p* + *t*, where *t* is the value shown on the *x*-axis. There is little consistency over time in reserves, as shown by the very weak correlation between *x*(*p*) and *x*(*p* + *t*) until *t* is very small. (*c*) The effect of the forage/rest threshold on the predation rate (dotted line), starvation rate (dashed line), and survivorship (solid line). If the animal has a low threshold it is likely to starve because it does not maintain high enough reserves to survive a run of bad luck when foraging. The number of individuals predated increases steadily as the maintained level of reserves increases. The highest survival is when the threshold is 19, as identified by the optimization procedure. The mortality cost of exceeding the optimal threshold by a given amount is much smaller than that of having a lower threshold than the optimum.

**Figure 2.**
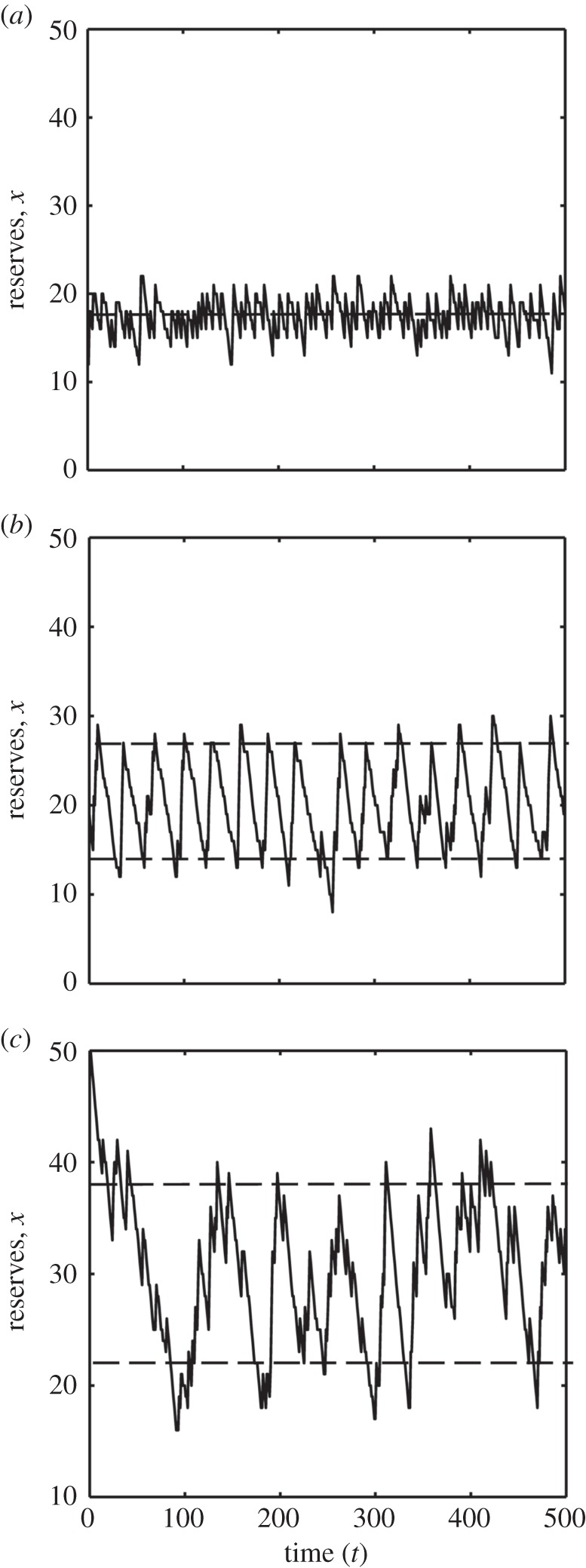
Simulation of a single individual following the optimal strategy showing reserves over time and upper and lower thresholds (dashed lines) at which the individual should switch for the (*a*) forage/rest model when switching does not involve predation risk, (*b*) forage/rest model with a switching predation risk, and (*c*) two-location model with a switching predation risk. In (*a*), there is a single set point, and the animal regularly switches between foraging and resting (it ‘dithers’). When switching is not risk free (*b*), the animal switches less often (dithering is reduced), but reserves fluctuate in a repeating pattern. In (*c*), the strategy mostly maintains reserves between the thresholds but there is some meandering between them, so the periods where the control system does not intervene are longer.

First, we consider the case where during one of the two activities the animal cannot find food nor be attacked by predators (*R*_L_ = 0, *R*_H_ = 0.4, *D*_L_ = 0, *D*_H_ = 0.0001, *D_C_* = 0), i.e. the choice is whether to rest or forage. In the case where there is no extra predation risk to switching, the optimal strategy is a single threshold that resembles a set point. While all individuals in a particular set of environmental conditions will have the same set point, there will still be variation in reserves around the set point among individuals due to stochasticity in finding food (figure 1*a*). This element of ‘luck’ in finding food means that there is also little within-individual consistency in energy reserves over time (figure 1*b*). Because there is no predation risk to switching, reserves fluctuate around the set point. That is, the animal appears to ‘dither’ between one activity and the other (figure 2*a*). We can alter this set point and find the associated rates of starvation and predation, and thus the cost of deviating from the optimal strategy (figure 1*c*). Survival is, of course, maximized at the optimal threshold, but it declines much more rapidly when the threshold is below the optimum than above it. This asymmetry is due to the starvation rate increasing very rapidly as the set point is lowered, whereas the predation rate only increases slowly as the set point is raised. Note that we have made no direct assumptions about these rates, but they follow from our assumptions about the stochasticity in the food supply, as follows. The length of time that the animal can survive without food is proportional to the level of reserves. The probability of failing to find any food during any period of time falls steeply as the length of the period increases. Therefore, the starvation rate will be a steeply declining function of the set point. The value of the set point will be where the increase in predation rate for a given increase in set point (i.e. the slope) is equal to the decrease in starvation rate for the same increase in set point [34]. Owing to the shape of the starvation rate function, this occurs where the starvation rate function is flattening out. Thus, the survival cost of storing more than the optimal amount of fat is much smaller than the cost of storing less.

This asymmetry in selective pressures occurs for all the sets of parameter values we have explored in our state-dependent model, in which foraging is stochastic and periods without food are due to a run of bad luck when searching for food in every time step. Periods of food shortage are therefore short and frequent, as assumed in the thrifty genotype hypothesis. However, animals including humans sometimes go through famine. In the electronic supplementary material, appendix B, we report the results when explicitly modelling famines of durations that are either exponentially distributed or normally distributed, and in the case where predation rate increases steeply with reserves. In all cases there is strong asymmetry in selective pressure. The shape of the starvation rate function also results in starvation being rare, with most mortality due to predation. In the electronic supplementary material, appendix C, we show that the rate of mortality from starvation is much smaller than the rate of mortality from predation in all these cases.

If the predation parameter when foraging (*D*_H_) is zero, the threshold level of reserves above which the animal should rest is very high, so that the risk of starvation is extremely small. As *D*_H_ increases, the threshold decreases (electronic supplementary material, figure D1a in appendix D). This is because it becomes increasingly important not to be vulnerable to predation and the animal has to accept a higher risk of starvation (electronic supplementary material, figure D1d). Previous theoretical work has led to the same prediction [25] which is supported by many observations of wild animals [35–39].

In many natural situations, food consumption may occur in a different location to resting (not least because foraging locations are often dangerous) which would result in a cost of travelling between the two locations. If there is a predation risk associated with switching (*D_C_* = 0.0001) two thresholds emerge and dithering is reduced, i.e. there are long bouts of both foraging and resting (figure 2*b*). This is because the time spent switching is now costly, so frequent switching should be avoided. As a result, we see the emergence of periods in which the animal feeds and periods in which it rests. Both thresholds decrease as the predation risk increases (electronic supplementary material, figure D1b), and they remain about the same distance apart.

### (b) Two-location model

**Figure 3.**
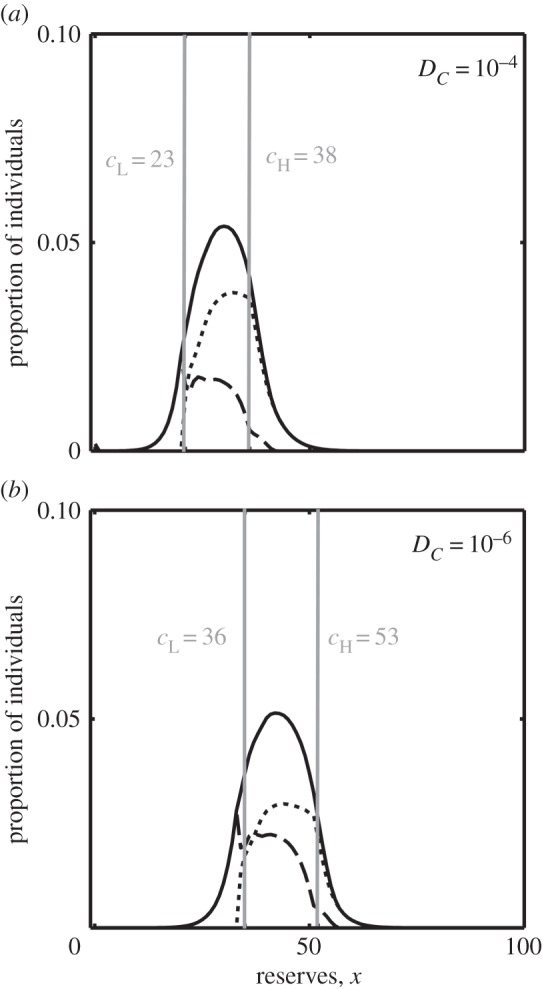
Distribution of reserves under (*a*) default predation levels (*D_C_* = 0.0001, *D*_L_ = 0.00001, *D*_H_ = 0.00003) and (*b*) very low predation levels (*D_C_* = 0.000001, *D*_L_ = 0.0000001, *D*_H_ = 0.0000003) in the two-location model. We show the probability distribution of surviving individuals in total (solid lines), during activity *L* (dotted line) and activity *H* (dashed lines). Grey lines indicate the locations of the two thresholds at the values shown. The reduction in predation risk, while not eliminating the upper threshold, causes an increase in maintained reserves but does not generate a right-skewed distribution.

A model in which the animal can both find food and be attacked by predators during both activities, but where one has both more food and more predators than the other (*R*_L_ = 0.1, *R*_H_ = 0.3, *D*_L_ = 0.00001, *D*_H_ = 0.00003, *D_C_* = 0.0001) results in a more realistic trajectory of reserves (figure 2*c*). During both activities reserves now fluctuate up and down, but during the *Low* activity reserves decline over time on average, whereas during the *High* activity they increase over time. Thus, both activities enable the animal to survive for some time, but by switching between them the animal can regulate its reserve levels. Increasing the risk of predation while foraging—while keeping the *relative* cost of switching constant (at 10× the cost of foraging)—again affects the optimal position of both thresholds (electronic supplementary material, figure D1c). Because the thresholds change in parallel, a reduction in the predation risk does not result in a skewed distribution of reserves, but the distribution is still symmetrical, only shifted to lower reserves (figure 3).

## 4. Discussion

The existing models of the control of adiposity all enjoy mixed empirical support, and engender much debate. However, none of them are sufficiently underpinned by an understanding of how evolution shapes behavioural strategies in animals, including humans, despite the widespread acknowledgement that an evolutionary approach could be crucial to understanding obesity [4,11,24]. Here we have, by means of a general evolutionary model, exposed the logic of these evolutionary arguments in an attempt to understand what assumptions are necessary to make them valid explanations for this important phenomenon.

### (a) Evaluating the set-point model

The concept of the set-point model is that the genotype (or early-life experience or epigenetic effects) determines one or more set points. Many approaches to understanding fat storage in animals have shown that a feeding strategy can be defined by a critical level of reserves below which the animal tries to gain more reserves [25,26]. This critical level is not necessarily a single fixed point, but may have multiple values for different times in the annual or seasonal cycles, or may vary in response to environmental changes. The critical level may increase [26] or decrease [25] as the availability of food increases, depending on the particulars of the food supply [25]. Thus, we expect that animals should have variable set points that adjust to current conditions. Such models (including ours) demonstrate that the observable distribution of reserves will be only partly determined by the set point(s), and partly by stochasticity in finding food. There have recently been attempts to assess obesity in domesticated, feral and semi-wild animal populations that seem to show trends towards obesity [40]. As these studies often have not measured individuals repeatedly (unlike studies on humans), it is difficult to use their data to assess the predictions of set-point models, because snapshots of a population at a single moment in time give no indications of the persistence of a particular body mass. It is important to note that the distribution of reserves in our model does not emerge from individual differences in foraging strategy, but from stochasticity in the environment affecting the reserves of individuals all following the same strategy. Therefore, despite the superficial similarity to the distribution of body mass in human populations, it is not clear that a simple set-point model applies, because people tend to maintain consistent body weights that differ between individuals [5]. It may be possible to understand the variation by assuming that individuals have different set points that are adapted to local conditions, both intrinsic (e.g. metabolism) and extrinsic (food and lifestyle). Whether it is possible to observe this depends on the relative size of within-individual variation around set points compared to the between-individual differences in set points, and how responsive set points are to changes in the environment.

It is likely that the concept of a set-point control system is unrealistically strict. In our model, the survival cost of negative deviations from the optimum reserves is greater than for positive deviations. This is also the case for the broad set of relationships between reserves and the starvation and predation risks that we study in the electronic supplementary material, appendix B. Thus, we can expect that the evolutionary pressure to maintain reserves at or above the optimum will be stronger than the pressure to prevent reserves increasing above the optimum. This provides an adaptive underpinning for existing models of feeding behaviour that have identified this asymmetry [41,42]. Overeating may occur as a result of two interacting influences. Firstly, if there is only weak selection not to be overweight, mutations that hinder the processes that prevent overeating may persist in a population. Secondly, the control systems for limiting weight gain could be easily overcome by external conditions such as highly rewarding tastes because, for example, the selective pressure to consume sweet foods where available has been strong [43]. Thus, in a complex environment with many factors affecting the strategy, we are likely to see individuals storing more fat than is optimal, owing to powerful environmental effects [44] overcoming (evolutionarily weak) limits.

We do not agree with Speakman [18, pp. 735] that environmental influences on the set point ‘effectively negate the utility of the set-point concept’ because a variable set point can still be a useful concept. Indeed, some authors have argued that a variable set point is a fundamental property of a control system ([45]; Hammel 1965 quoted in Cabanac 2006, p. 1341, [46]). Such a set point would alter in response to changes in the food supply and/or, perceived risk of predation in order to maintain an optimal level of reserves for the current conditions [12]. Speakman and colleagues present evidence that fat storage responds to seasonal changes in food availability [47]. In social animals, predation risk and access to food both depend on social status, and there is abundant evidence that fat storage responds to social status [48–50], as expected from the variable set-point concept. We conclude that development of variable set-point models is likely to help understand patterns of obesity across seasons, ages, and genders in humans (e.g. [51,52]).

### (b) Evaluating the dual intervention point model

In our view, it is not sufficient for an evolutionary explanation to posit a mechanism without providing a robust account of the adaptive value of that mechanism. That is, the dual intervention point model is incomplete without an explanation for why the system should be indifferent to all the states between the thresholds. In the dual intervention point model, the animal acts to avoid starvation if the lower threshold is reached, and acts to avoid predation if the upper threshold is reached. In between the thresholds, the animal is indifferent to the level of reserves. The problem with this view is that the level of reserves partly determines the likely amount of reserves in the near future, and so influences the future risk of starvation and predation. In the case where foraging is somehow costly and doing nothing means reserves will tend to decrease over time, the animal should try to sustain its reserves just above the lower threshold. In any case, the animal should just have a single set point that minimizes the total mortality rate taking into account future states. Thus, the dual intervention point model is incomplete without positing an adaptive reason that any system should be indifferent to the level of reserves over any part of the range. In order to provide a logical model of the drifty gene hypothesis, we have posited one reason for two thresholds: that the *response* to reaching a threshold is somehow costly and so the system should avoid switching too often. This does not mean that the system should be indifferent to gaining or losing weight as Speakman suggests [18]; in fact, the decision to continue doing the current activity is strictly optimal. It does however mean that among individuals of equal weight, some will be gaining weight and some losing weight, and at the population level this may look like people are indifferent.

Our model is inspired by abundant literature, especially in the field of behavioural ecology, on the starvation–predation trade-off [25,26,29,53,54]. A development of foraging models uses two thresholds to avoid costly dithering [55–57], which leads to more realistic behaviour and was evoked in the justification for the dual intervention point model [23]. Here, we have characterized the thresholds as controlling the change between activities, where the two activities could also reflect feeding on different foods or even eating differing amounts. We agree with Speakman [23] that the fat storage system evolved to trade-off the opposing risks of starvation and predation. We do not agree about how the levels of these thresholds are determined. Speakman [58] states ‘Considerable research suggests that this fundamental balance of risks of starvation keeping body masses up (i.e. setting the lower intervention point) and risks of predation keeping body masses down (i.e. setting the upper intervention point) is a key component of body mass regulation in many wild animals—including both mammals and birds (e.g. [26,30,59,60])’. However, none of these cited papers provide any evidence that any animal has two intervention points, only that there are two opposing selective pressures on body mass.

We have shown that it is optimal to have two thresholds if there is a cost of switching. If there are, then the level of both the upper and the lower thresholds depends on both the starvation risk and the predation risk. A decrease in predation risk in ancestral humans would have led to an increase in the level of both thresholds (electronic supplementary material, figure D1). With the lower threshold set at a higher value, the minimum level of fat should be much higher than it was before, while the range of fat should remain approximately unchanged (figure 3). These results illustrate the fact that the drifty gene hypothesis [58] is based on the implicit assumption that predation risk and starvation risk are present only for some levels of energetic reserves. That is, predation only occurs if reserves are above the upper threshold, and the risk of starvation is only greater than zero below the lower threshold. The assumption is (perhaps unintentionally) made more explicit by the statement ‘[the lower threshold] probably allows the animals to survive any minor food security crisis they may normally encounter in the wild’ [58, pp. 7]. Actually, both predation risk and starvation risk are greater than zero at all values of reserves, and thus both thresholds should be sensitive to both sources of mortality. In any case, it is far from clear that the development of weaponry and the control of fire wholly eliminated the risk of predation on humans [61,62]. Furthermore, being overweight has other potential negative consequences for the animal, such as reduced ability to hunt and to compete for mates, which would not have been eliminated by the technological developments of ancestral humans. However, this is perhaps not a significant issue because it is sufficient that predation risk has declined greatly, even if not to zero.

Speakman [22] suggests that the dual intervention point model is supported by data on small rodents [63–65]. However, these studies present no evidence that there is more than one set point, instead showing an effect of seasonality. That is, they provide evidence that animals behave as though they have a variable set point. Speakman [58] used a genetic model to predict the distribution of 'body mass index (BMI) in modern humans given the drifty gene hypothesis and claims there is a good fit to observed distributions. In the electronic supplementary material, appendix D, we show that the predictions of this genetic model are changed when both the lower and upper thresholds are allowed to drift. The changed predictions do not match observation, meaning that the distribution of body masses in human populations in fact does not support an account in which both thresholds drift. In any case, while an argument based on adaptive explanations may give a description of the behaviour of animals, it has no explanatory power unless it is logical, and we have shown that the dual intervention point model is not.

### (c) The role of famines

There has been much debate over the role of famines in determining the fat storage strategy of modern humans. Some researchers [22] have argued that famines were insufficiently common to drive the evolution of thrifty genes, because famines are relatively rare and few individuals die in famines. On the other hand, it has been pointed out that if a thrifty genotype is advantageous then all people should be obese [4]. Other authors [3] have argued that mortality is unimportant in comparison to the adverse effect of food shortage on reproduction, which will be greater for lean individuals. The role of famines may be overstated because as food supplies fluctuate, the main function of fat may be to buffer short-term shortfalls in food intake [5]. This may be more applicable to a hunter–gatherer existence, while after the agricultural revolution famines may have been more common [13], although recent evidence suggests only weak associations between crop yields and survival or fecundity [66]. Arguments for the drifty genes hypothesis do not invoke ‘periods of famine, but … periods of a few days when the individual failed to secure food’ [57, pp. 8], which is exactly how we have conceived the food supply in our main model. That is, animals should be adapted to manage their fat reserves against runs of bad luck in foraging, as well as times of complete absence of food or when foraging is impossible.

Whichever is the case, we have shown here that low mortality from starvation does not imply that the risk of starvation has been unimportant in determining feeding strategies. In all realistic parametrizations of our models (main text and the electronic supplementary material, appendix C), the starvation rate was small in comparison with the predation rate (as in previous models, e.g. [12,25]), yet the risk of starvation has a powerful influence on the position of the optimal threshold, as shown by the change in threshold when we alter the predation risk. Note that when the risk of a food shortage is zero, the optimal level of reserves is as small as possible to stay alive (e.g. unity). Hence, increasing uncertainty of the food supply or rate of famines tends to increase reserves and so increase the predation rate. It is even possible that in some circumstances the probability of starvation may increase as food availability increases [12,34]. Thus, the observation that most mortality even during famines is not from starvation [4] is not evidence against the role of the risk of starvation in determining fat storage strategies. We conclude that the magnitude of the mortality during food shortages, including famines, is uninformative about selective pressures, and so further debate on this issue is unlikely to help assess the validity of the evolutionary explanations for obesity.

In a stochastic environment such as the one assumed by the dual intervention point model, energy reserves may appear to wander freely between the two thresholds. The observed consistency in the body weights of many people over time contradicts this prediction of the model. However, the meandering weight of dieting obesity-susceptible people [10] may be explained by this model: dieting would be successful until the lower intervention point is reached, whereupon the urge to stop dieting will be strong. Thus, we do not suggest that a model with two thresholds cannot help us to understand weight control, but it should not only be properly founded in evolutionary theory but also be significantly more sophisticated, taking into account changes in the environment.

The evolutionary approach clearly has the potential to improve our understanding of human behaviour, but it may be that obesity cannot be modelled in the simple way that has been adopted by us and others. The modern environment is geared to exploit errors in the mechanisms that control food intake [43]. There is clearly a need to model these mechanisms realistically, but since the mechanisms often cannot be directly assessed, it is critical that such models make sense *in the light of evolution* [67]. We have shown here that such an approach may be the most appropriate way to achieve a sufficient understanding of the causes of obesity in order to reverse the advance of this important health problem. We believe that progress in reducing obesity could result from a sophisticated assessment of how previously adaptive behavioural mechanisms might be maladaptive in the modern world [68]. For instance, the amount of energy consumed is determined not directly by the size of fat stores, but by proximate factors that are intrinsic to the animal (e.g. circulating hormones) and extrinsic (e.g. food stimuli). If we can characterize the interactions among these factors in an evolutionary model, we can better predict how the modern environment interacts with our inherited eating control mechanisms to cause obesity.

## Supplementary Material

Online Appendices

## Data accessibility

The Matlab code used to generate the results is available on request from the authors.
