# Supplementary material for: Fatness and fitness: exposing the logic of evolutionary explanations for obesity
Source: Proc Biol Sci. 2016 Jan 13;283(1822):20152443. doi: 10.1098/rspb.2015.2443 (PMC4721100; doi:10.1098/rspb.2015.2443)

**ONLINE APPENDICES**

Higginson A. D, McNamara JM, Houston AI (2016) Fatness and fitness: Exposing the logic of evolutionary explanations for obesity. *Proc Roy Soc Lond B*

**APPENDIX A**

Here, we provide a complete description of the model implementation. Our optimization criterion follows that in McNamara (1990) and can be described as follows. At a decision epoch reserves are assumed to take values in the range $x=0, 1, 2, \cdots,s$, and the forager is currently doing activity *A*, which can be either low (*A* = *L*) or high (*A*= *H*). At each decision epoch the animal chooses whether to stay (α=0) or switch (α=1). The decision α is allowed to depend on both the energy reserves of the animal and the current activity. A strategy $\pi$ specifies this dependence; under $\pi$ the decision when reserves are *x* and the activity is *A* is denoted by$\alpha_{\pi}(x,A)$. Let $S_{\pi}(x,A,t)$ be the probability that an animal that follows strategy$\pi$ survives until time *t* given that its reserves are *x* and it is doing activity *A* at time 0. Then it follows by standard Markov chain results that there exists a function of state $h_{\pi}(x,A)$and a constant $\varphi(\pi)$ such that

$S_{\pi}(x,A,t) \sim h_{\pi}(x,A)(\varphi(\pi))^{t}\text{ as }t\to\infty$. (A1)

The function *h* represents the transitory effect of initial conditions. The constant $\varphi(\pi)$ is the probability that an animal that is alive at some large time *t* is still alive at *t*+1. Thus, if$\varphi(\pi_{1})<\varphi(\pi_{2})$ for two strategies$\pi_{1}\text{ and }\pi_{2}$, the probability of surviving until time *t* is greater under $\pi_{2}$ than under $\pi_{1}$ for all sufficiently large *t*; i.e. $S_{\pi_{2}}(x,A,t)>S_{\pi_{1}}(x,A,t)$ for all sufficiently large *t*. Motivated by this we define a strategy $\pi*$ to be optimal if

$\varphi(\pi*)=\max_{\pi} \varphi(\pi)$. (A2)

If the decision between time *t* and *t* + 1 is α, then the probability of finding an item of food during this time interval is (1-α)*R_A_*, because the animal cannot find food when switching. Food items are of three types (type *j* = 1, 2 or 3) with relative abundance ρ*_j_* (Σρ *_j_* = 1), and provide a mean reward of energetic value *r* and variability σ. The value of the food types *r_1_*, *r_2_*, *r_3_* are *x-σ, x, x+σ* respectively. In each time step the forager pays an energy cost *m*(*x*) to meet its metabolic needs that increases with reserves. The probability the animal is not killed by a predator before the next decision epoch is $1-\mu(x,\alpha,A)$. Thus if the animal has reserves *x* at time *t* its reserves at time *t* + 1 are either $x-m$, $x+r_{1}-m$, $x+r_{2}-m$ or $x+r_{3}-m$, and these possibilities occur with probabilities $[1-\mu(x,\alpha,A)][\alpha+(1-\alpha)(1-R_{A})]$, $[1-\mu(x,\alpha,A)](1-\alpha)R_{A}\rho_{1}$, $[1-\mu(x,\alpha,A)](1-\alpha)R_{A}\rho_{2}$ and $[1-\mu(x,\alpha,A)](1-\alpha)R_{A}\rho_{3}$ respectively. If the change in reserves results in the new reserves being zero or below, the animal is assumed to have died of starvation. If the new reserves would have been greater than the maximum value of *s*, then reserves are taken to be *s*.

Given these ingredients, the dynamic programming operator *T** can be expressed follows. Let *V* be a function, $V\left(x,A\right)$, of energy reserves *x* and activity *A* satisfying $V\left(0,A\right)=0$. Then $T *V$is a new function of reserves and activity that satisfies $(T *V)\left(0,A\right)=0$for all *A* and$(T*V)\left(x,A\right)=\max_{\alpha} H(x,A;\alpha,V)$ for *x* > 0 and all *A*, where

$H(x,A;\alpha,V)=\left[1-\mu\left(x,\alpha,A\right)\right]\left(\begin{matrix} \{\alpha+(1-\alpha)(1-R_{A})\}\left[\left(1-\alpha\right)\cdot V\left(\max(x-m,0),A\right)+\alpha\cdot V\left(\max(x-m,0),\neg A\right)\right] \\ +(1-\alpha)R_{A}\sum_{j=1}^{3} \rho_{j}\cdot V\left(\min\left(x+r_{j}-m,s\right),A\right) \end{matrix}\right)$(A3)

where *¬A* is the other activity.

To find the optimal strategy, we define a sequence of functions $V_{0}, V_{1}, V_{2},\cdots$ iteratively as follows. Initially set $V_{0}\left(0,A\right)=0$ for all *A* and $V_{0}\left(x,A\right)=1$ for all $x>0$ and all *A*. Given $V_{k}$, set $V_{k+1}=T *V_{k}/\left|T *V_{k}\right|$, where the norm, $\left|V\right|$, of a function *V* is given by $\left|V\right|=\max_{x,A} \left|V(x,A)\right|$. Then the sequence of functions converges pointwise to a limit $V *=\lim_{k\to\infty} V_{k}$ [McNamara, 1990]. In computations, convergence was judged to have occurred when $\left|V_{k+1}-V_{k}\right|<10^{-6}$, which typically happened within 100 iterations. Any strategy $\pi*$ satisfying

$H(x,A;\alpha_{\pi*}(x,A),V*)=\max_{\alpha} H(x,A;\alpha,V*)\text{ for all }x>0\text{ and all }A$ (A4)

necessarily satisfies equation (A2), and is hence optimal [McNamara, 1990].

**Reference**

McNamara, J. M. 1990 The policy which maximises long-term survival of an animal faced with the risks of starvation and predation. *Adv Appl Prob*, **22**, 295-308.

Table A1: Parameters in the model and their default values.

| Symbol | Parameter | Forage/rest model | Two-location model |
| --- | --- | --- | --- |
| *s* | Maximum reserves | 100 | 100 |
| *m* | Metabolic cost | 1 | 1 |
| *r* | Mean energy of food items | 5 | 5 |
| *σ* | Variability in energy of food items | 1 | 1 |
| *R_L_, R_H_* | Probability of finding food item during low (*L*) and high (*H*) activity | 0, 0.4 | 0.1, 0.3 |
| *D_L_, D_H_* | Maximum predation risk during low (*L*) and high (*H*) activity | 0, 0.0001 | 0.00001, 0.00003 |
| *D_C_* | Maximum predation risk when switching | 0 *or* 0.0001 | 0.0001 |

**APPENDIX B: Asymmetry in selective pressures**

Here we show that, under realistic conditions, the increase in mortality as reserves decrease below the optimum will be greater than the increase in mortality as reserves increase above the optimum. Thus, the selective pressure to avoid being underweight will usually be stronger than the selective pressure to avoid being overweight. Initially we assume that predation risk increases linearly with reserves, because increasing fat load reduces the ability to evade predators and/or increasing metabolic costs cause the animal to spend more time foraging for food and therefore exposed to predators. The risk of starvation is assumed to depend on the likelihood that a shortage of food occurs and the duration of such a shortage. Firstly, we show that if the durations of famines are exponentially distributed, which will mean that the rate of starvation decreases by a constant with each additional unit of reserves, then it is always the case that the fitness costs have negative skew. Other distributions are not tractable in a without assuming the form of the relationships, so we go on to assess the conditions we expect negative skew in more situations by exploring the impact of some reasonable forms.

**General case**

We denote the probability distribution of famine durations as *f*(*t*). During a famine the animal loses reserves at unit rate. The probability that the animal dies given a famine has occurred is $\int_{x}^{\infty} f(t)dt$, so the rate of starvation at reserves *x* is

$S(x)=\lambda\int_{x}^{\infty} f(t)dt$ (B1)

where λ is the rate with which famines occur. Then

$S'(x)=-\lambda f(x)$ (B2a)

$S''(x)=-\lambda f'(x)$ (B2b)

$S'''(x)=-\lambda f''(x)$(B2c).

Assume that the rate of predation linearly increases with *x* with coefficient *k*:

$P(x)=kx$ (B3)

So the total mortality rate is

$T(x)=\lambda\int_{x}^{\infty} f(t)dt+kx$. (B4)

Given (B2a) and (B3) we see that at the optimal level of reserves (*x**)

$\lambda f(x*)=k$ (B5)

and if there is an optimal level of reserves *f*(*x**) is a minimum (B2b) implies

$-\lambda f'(x*)>0\Rightarrow f'(x*)<0$ . (B6)

Now,

$T'''(x)=S'''(x)=-\lambda f''(x*)$ (B7)

so,

$T'''(x*)<0\Leftrightarrow f''(x*)>0$ (B8)

which is always the case when *f*(*x*) is exponentially distributed. If *f*(*x*) is normally distributed then (B8) holds when *x** is greater than the sum of the mean and standard deviation of *f*(*x*) i.e. if the animal survives most famines.

**Specific forms of relationships**

Next, we show that we expect negative skew in the exponential case for a specific functional form.

***Model 1***

An exponential form of famine duration might be expected if there is a constant probability over time that the food supply will recommence. The starvation rate can be given by:

$S(x)=ae^{-bx}$ (B9)

where *a* and *b* are both positive numbers so that *S*(*x*) decreases as *x* increases.

The predation rate increases linearly with reserves (*x*):

$P(x)=kx$ (B10)

where *k* is a positive number. The total mortality rate *T*(*x*) is maximised or minimised when

$0=k-\frac{ab}{e^{bx}}$ (B11)

so the level of reserves at a maximum or minimum is

$x*=-\frac{\log\left(\frac{k}{ab}\right)}{b}$ . (B12)

We find the value of the second derivative at *x**

$\frac{\partial^{2}T}{\partial x^{2}}=\frac{b^{2}a}{e^{bx}}$ (B13)

i.e.

$\frac{\partial^{2}T}{\partial x^{2}}(x=x*)=bk$ (B14)

which is positive, so the stationary point is a minimum and this level of reserves (*x**) is the optimal level. The third derivative is

$\frac{\partial^{3}T}{\partial x^{3}}=-\frac{b^{3}a}{e^{bx}}$ (B15)

Which at *x** has the value

$\frac{\partial^{3}T}{\partial x^{3}}(x=x*)=-b^{2}k$ , (B16)

which is negative, meaning that the magnitude of the decrease in *T*(*x*) as *x* decreases from *x** is greater than the magnitude of the decrease in *T*(*x*) when *x* increases from *x**. Thus, having lower reserves than is optimal is more costly than having higher reserves than is optimal. An example parameterisation is shown in Figure B1a & b.

***Model 2***

If the durations of interruptions to the food supply are normally distributed, as may be the case for famines, the relationship between reserves and the rate of starvation may follow a logistic function. The starvation rate is

$S(x)=a\left(\frac{1}{1+e^{b(x-c)}}\right)$ (B17)

where *a* is proportional to the frequency of famines, *b* is proportional to the variance in famine duration, and *c* is the level of reserves that would allow the animal to survive a famine of mean duration. An example parameterisation is presented in Figure B1c & d. Again there is a minimum at positive *x*, but also a maximum, so having a small amount of reserves results in a higher mortality rate than no reserves, because the predation rate is higher but the starvation rate is no lower. The level of reserves at the stationary points are

$x*=c+\frac{1}{b}\ln\left(\frac{ab\left[1\pm\sqrt{1-\frac{4k}{ab}}\right]}{2k}-1\right)$ . (B18)

Note that *x** has two values, which are real only if

$ab>4k$. (B19)

Since the optimal strategy is to minimise mortality rate, we can ignore the *x** associated with a maximum. The value of the second derivative at a minimum is

$\frac{\partial^{2}T}{\partial x^{2}}(x*)=bk\sqrt{1-\frac{4k}{ab}}$ . (B20)

The third deriviative is

$\frac{\partial^{3}T}{\partial x^{3}}=-\frac{ab^{3}e^{b(c-x)}\left(e^{2b(c-x)}-4e^{b(c-x)}+1\right)}{\left(e^{b(c-x)}+1\right)^{4}}$ (B21)

which at the minimum has the value

$\frac{\partial^{3}T}{\partial x^{3}}(x*)=\frac{6bk^{2}}{a}-b^{2}k$ (B22)

Which is negative if:

$ab>6k$ . (B23)

This is the condition for a decrease in reserves from the optimum to be more costly than an increase from the optimum. From (B19) and (B23) we see that there is a real solution for an increase in reserves from the optimum to be more costly than a decrease only if

$4k<ab<6k$ (B24)

which is a fairly narrow condition and is unlikely to occur in the real world (Figure B2). Thus, it is likely that in most situations that the selective pressure to avoid becoming underweight will be stronger than the selective pressure to avoid being overweight.

***Model 3***

It is possible that predation risk increases with reserves at an accelerating rate, if being overweight reduces the ability to evade or escape from predators. Assume that both *S*(*x*) and *P*(*x*) are exponential such that predation risk increases with reserves at an accelerating rate:

$S(x)=ae^{-bx}$, (B25a)

$P(x)=ce^{dx}$, and (B25b)

$T(x)=ae^{-bx}+ce^{dx}$ (B25c)

where *a*, *b*, *c*, *d* are positive numbers. An example parameterisation is shown in Figure B1e & f. The value of reserves at the stationary point is

$x*=\frac{\log\left(\frac{ab}{cd}\right)}{b+d}$ . (B26)

The value of the second deriviative is

$\frac{\partial^{2}T}{\partial x^{2}}(x*)=\frac{ab(b+d)}{\left(\frac{ab}{cd}\right)^{\frac{b}{b+d}}}$ (B27)

which is positive, indicating that the stationary point is a minimum and so *x** is the optimal level of reserves. The value of third derivative is

$\frac{\partial^{3}T}{\partial x^{3}}(x*)=-\frac{ab(b^{2}-d^{2})}{\left(\frac{ab}{cd}\right)^{\frac{b}{b+d}}}$ (B28)

which is less than zero if:

$d<b$. (B29)

Thus, which selective pressure is greatest depends on the slope of their increases over time, and not on the magnitude of risk of starvation and predation. Thus, a reduction in reserves from the optimum is likely to be more costly than an increase unless predation rate is very strongly influenced by body mass. This is possible in flying animals that may have reduced take-off acceleration, but is unlikely in terrestrial animals.

**Figure B1**: Example parameterisations of the functional forms analysed in Appendix B showing (a, c, e) mortality rates and (b, d, f) the absolute value of the slope (i.e. first derivative) of the mortality rates with respect to reserves (*x*). The starvation rate (dotted lines) decreases with increasing reserves (x), whereas the predation rate (dashed lines) increases with increasing reserves. Total mortality rate (solid lines) is therefore minimised at some intermediate reserves. (a, b) ***Model 1***: $S(x)=1\cdot e^{-0.1x}$, $P(x)=0.01x$, (c, d) ***Model 2***:$S(x)=1\cdot\left(\frac{1}{1+e^{0.2(x-20)}}\right)$, $P(x)=0.01x$, (e f) ***Model 3***: Both starvation and predation rate follow exponential functions $S(x)=1\cdot e^{-0.1x}$, $P(x)=0.05e^{0.05x}$.


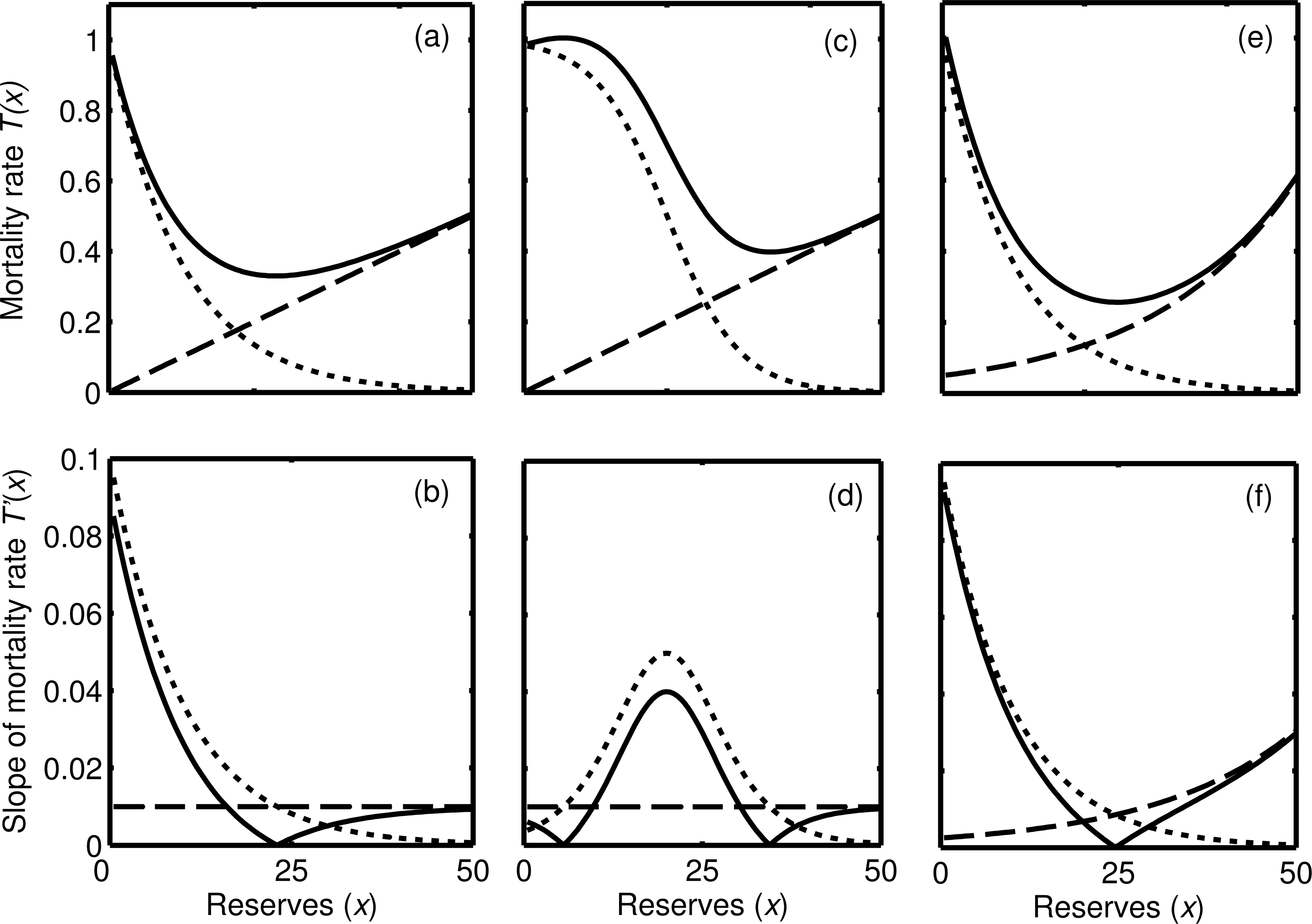


**Figure B2**: Effect of the parameter values in the condition where the effect of increasing reserves on decreasing risk of starvation follows a logistic function (e.g. the duration of famines follows a normal distribution), and predation rate increases linearly with reserves. The panels show, for each combination of parameters, the sign of the third derivative at the optimal level of reserves *x** (– indicates that having lower reserves than *x** is more costly than having higher reserves than is optimal, + indicates the opposite), and which source of mortality is the larger (*P* indicates more individuals die from predation at *x**, *S* indicates that more individuals die from starvation at *x**). White areas are combinations where there are no real solutions (i.e. optimal reserves is negative or positive infinity). (a) frequency of famines *a* against magnitude of predation risk *k*, both on a log scale, (b) level of reserves that would enable survival of a famine of mean duration *c* against magnitude of predation risk *k*, the latter on a log scale, (c) frequency of famines *a* against variation in famine duration *b*, the former on a log scale. Since it is known that most individuals in most systems die from predation (see main text), parameter combinations where more individuals die from starvation are probably unrealistic. The parameter combinations that give real solutions are almost all (–,P), i.e. predation rate is higher than starvation rate and the selective pressure against having lower than optimal reserves is stronger than that against having higher than optimal reserves. Thus, we conclude that in most realistic conditions, animals will have evolved weak controls against being overweight.


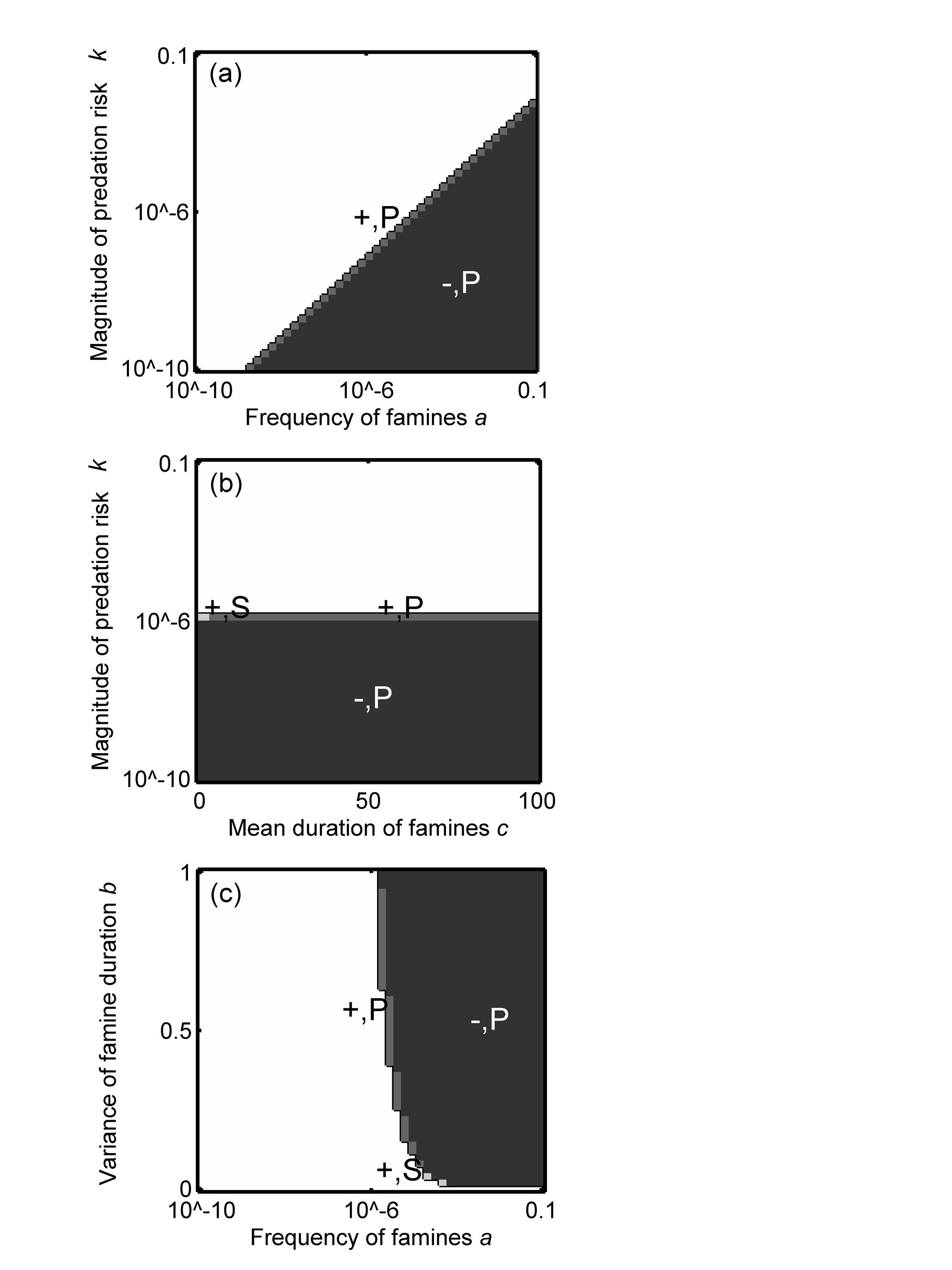


**APPENDIX C: Source of mortality**

Here we find the conditions under which the predation rate will exceed the starvation rate for the three models explored in Appendix B, that assume different functions for the relationship between reserves and the starvation and predation rates.

**Model 1**

The starvation rate can be given by:

$S(x)=ae^{-bx}$ (C1)

where *a* and *b* are both positive numbers so that *S*(*x*) decreases as *x* increases.

The predation rate increases linearly with reserves (*x*):

$P(x)=k(c+x)$ (C2)

where *c* and *k* are positive numbers. The total mortality rate *T*(*x*) is maximised or minimised when

$0=k-\frac{ab}{e^{bx}}$ (C3)

so the level of reserves at a maximum or minimum is

$x*=-\frac{1}{b}\log\left(\frac{k}{ab}\right)$. (C4)

The ratio of the predation rate to the starvation rate at optimal reserves is

$bc-\log\left(\frac{k}{ab}\right)$ (C5)

which is greater than unity when

$k<ab\cdot e^{bc-1}$ . (C6)

The effect of each parameter for example values are shown in Figure C1a, d, g. Some effects of parameters are counter-intuitive for most of the parameter space: *P*(*x**):*S*(*x**) increases – indicating relatively more predation compared to starvation – as the risk of a food shortage *a* increases, the duration of famines *b* increases, and the minimum vulnerability *c* increases, and decreases as the dependence of predation on reserves *k* increases. To understand these results, first consider that *x** decreases as *k* increases. Now, *S*(*x**) increases linearly with *k* and *P*(*x**) increases at decelerating rate over reasonable values of *k.* This occurs because the optimal level of reserves is always at the point where the starvation rate increases steeply (Appendix B). Hence, an increase in *k,* reducing *x** will tend to increase *S*(*x**) more than *P*(*x** ). The asymmetry in the slopes around *x** explain the direction of the effects of other parameters show in Figure C1 in a similar manner.

**Model 2**

If the durations of interruptions to the food supply are normally distributed, as may be the case for rare but long famines, the relationship between reserves and the rate of starvation may follow a logistic function. Assume that the starvation rate is

$S(x)=a\left(\frac{1}{1+e^{b(x-c)}}\right)$ (C7)

where *a* is proportional to the frequency of famines, *b* is proportional to the variance in famine duration, and *c* is the level of reserves that would allow the animal to survive a famine of average duration. The predation rate increases linearly with reserves (x):

$P(x)=kx$. (C8)

In this case,

$\frac{P(x*)}{S(x*)}=\frac{b(J+1)}{2}\cdot\left(c+\frac{1}{b}\log\left[\frac{ab}{2k}(J+1)-1\right]\right)$ (C9)

where $J=\sqrt{1-\frac{4k}{ab}}$. Examples of equation (C9) are plotted in Figure C1b, e, h. The effects of each parameter are qualitatively unchanged from *Model 1* for the ranges of parameter explored, and *P*(*x** ) is nearly always greater than *S*(*x**) .

**Model 3**

If both *S*(*x*) and *P*(*x*) are exponential such that predation risk increasing with reserves at an accelerating rate:

$S(x)=ae^{-bx}$, (C10a)

$P(x)=ke^{cx}$ (C10b)

where *a*, *b*, *c*, *d* are positive numbers, then

$\frac{P(x*)}{S(x*)}=\frac{b}{c}$. (C11)

Once again, for the reasonable values of the parameters in Figure C1c, f, i, the predation rate almost always exceeds the starvation rate, often up to 10×. Furthermore, the proportion of individuals dying from starvation actually declines as the frequency of famines increases. Thus, the starvation rate is not a good metric of the magnitude of the selective pressure from a source of mortality.

Figure C1: Ratio of predation rate *P*(*x**) to starvation rate *S*(*x**) at optimal reserves *x** for ranges of the parameters shown on the x-axes and legends for the three models described in the Appendix C. In all cases and across all ranges, the rate of predation far exceeds the rate of starvation. Furthermore, the relative rates of starvation do not relate in a clear way to the magnitude of the potential risks of mortality, being highly non-linear or flat.


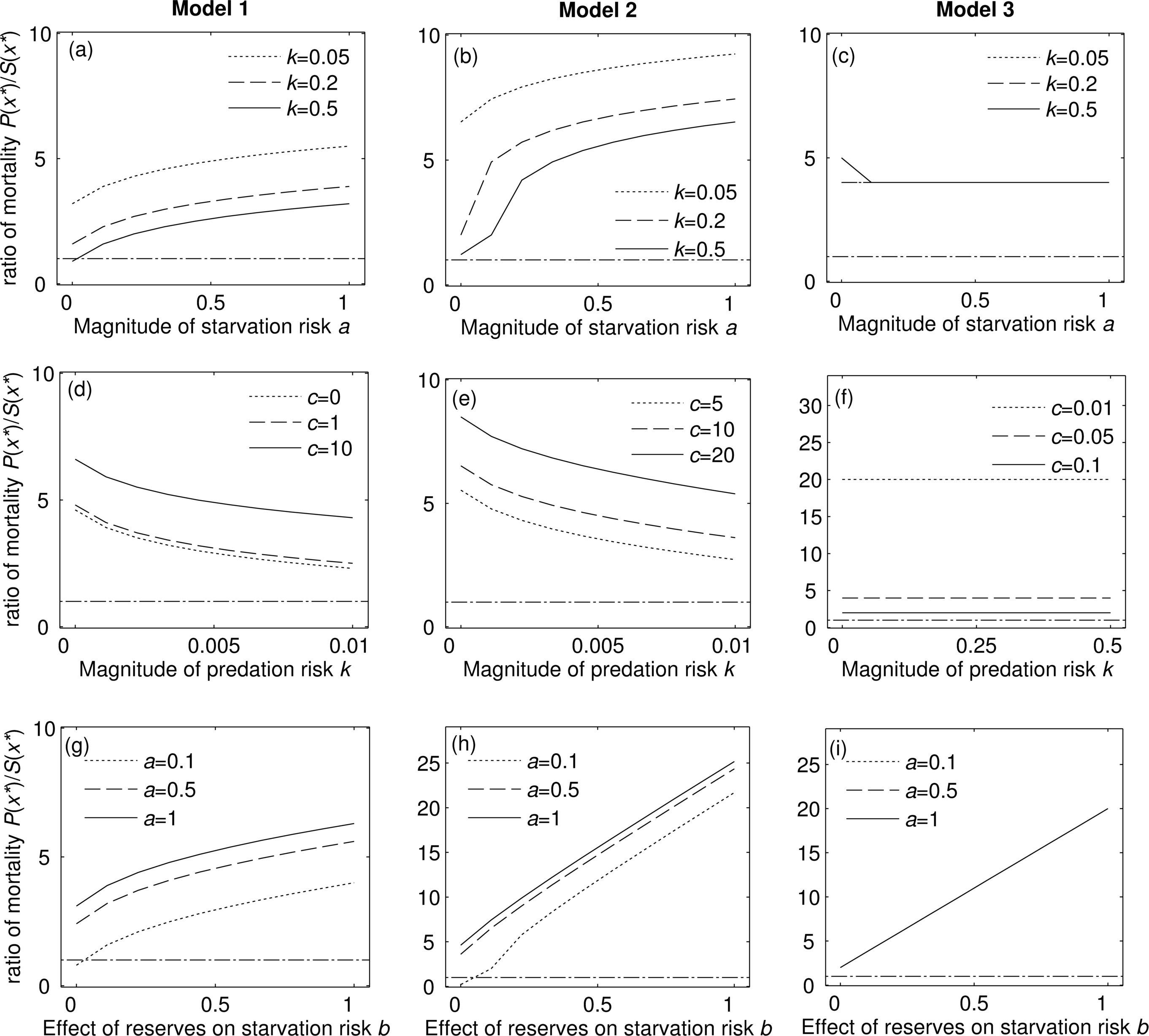


**APPENDIX D: Additional results**

**Effect of predation risk**

**Figure D1**: Effect of overall predation risk for the (a, d) forage/rest model with no switching cost (*D_H_* shown on *x*-axis; *D_L_* = 0, *D_C_* = 0), (b, e) forage/rest model with a switching cost (*D_H_* shown on *x*-axis; *D_L_* = 0, *D_C_* = *D_H_*), (c, f) two-location model with a switching cost (*D_C_* shown on *x*-axis; *D_L_* = 0.1*D_C_*, *D_H_* = 0.3*D_C_*). We show (a, b, c) optimal thresholds *c*(*L*) (dashed lines) and *c*(*H*) (solid lines) and (d, e ,f) the proportion of individuals killed by predation (solid lines) and starvation (dashed lines) during 1000 time steps


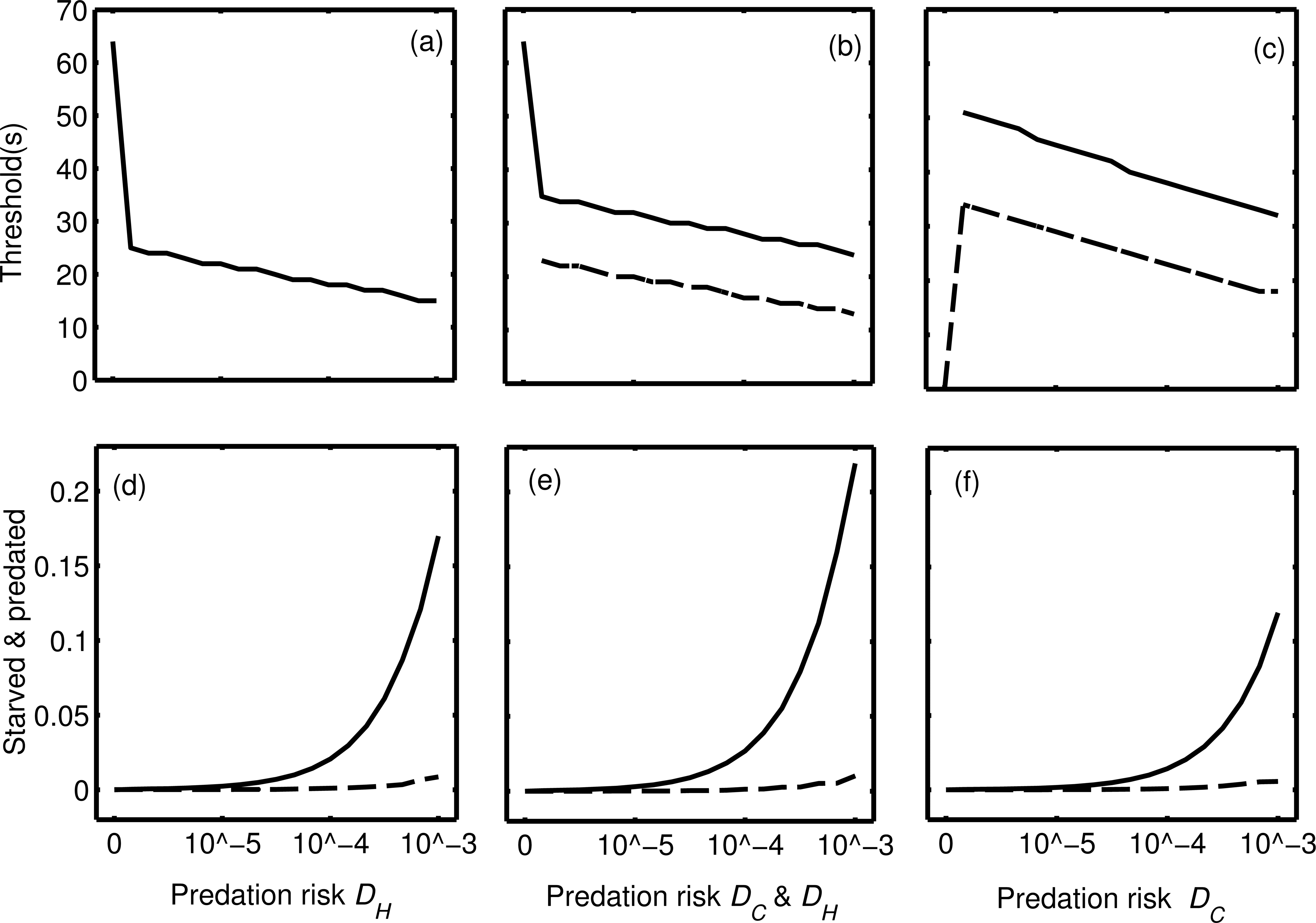


**Distribution of BMI**

Speakman [2007] used a genetic model to predict how the removal of predation might have influenced the body mass index (BMI) of a population via drift in the genes coding for a threshold (‘intervention point’). This model assumes that (i) only the upper threshold will have drifted and (ii) that all individuals have a BMI equal to their genetically-encoded upper threshold. Here, we assess how the predictions of this genetic model are altered when assumptions (i) and (ii) are relaxed: i.e. (i) if an individual’s BMI takes a normally distributed random value between their lower and upper thresholds, and (ii) if both the lower and upper thresholds may have drifted.

We will briefly summarize Speakman’s analysis before describing how we have further developed it. Speakman [2007] assumed the thresholds are determined by few genes with large effects and that each mutation in these genes causes an increase or decrease in the thresholds by eight BMI units. He further assumed that the numbers of mutations causing positive and negative changes are both random variables following a Poisson distribution with a mean of 1.5. The threshold of a given genotype is then simply the ancestral value plus eight BMI units multiplied by the number of positive mutations minus eight BMI units multiplied by the number of negative mutations. Speakman [2007] took a starting point of mean BMI of 20 from hunter-gathering peoples in Namibia [Kirchengast 1998] and studied the predicted distribution of BMI if only the upper threshold changes and all individuals have masses at their upper threshold, assuming that genotypes that decreased the threshold would have been eliminated by selection.

The process is more complex when both thresholds may change. Firstly, we needed to estimate what the lower and upper threshold might be for hunter-gatherers. Kirchengast [1998] reports a mean BMI of 19.55 with standard deviation of 2.67. Assuming that people maintain their BMI between the lower and upper thresholds 95% of the time and that BMI follows a Gaussian distribution we can infer that the lower threshold is 19.55–2×2.67 (=14.21) and the upper threshold is mean 19.55+2×2.67 (=24.89). Note that this upper threshold is close to the boundary of what is considered ‘overweight’ [Flegal et al 2002]. We then assume that both thresholds can have between zero and six positive and between zero and six negative mutations, with the probability of each number taken from a truncated Poisson distribution with a mean of 1.5, and only those mutations that reduce the lower threshold are assumed be selected against (i.e. removed from the gene-pool). We can then calculate the distribution of modern BMI based on the new thresholds using simple arithmetic with the conditional probabilities of the numbers of positive and negative mutations in both thresholds.

We successfully replicated the predictions shown in Speakman [2007] (white bars in Figure D2a). There are three new possibilities that we can compare. (1) Both thresholds may have drifted and all individuals have a BMI equal to whichever threshold is higher (black bars in Figure D2a). (2) Individuals’ masses are normally distributed between the thresholds of each genotype when both thresholds may have drifted (black bars in Figure D2b). (3) Individuals’ masses are normally distributed between the thresholds of each genotype, but the lower threshold has remained fixed for whatever reason Speakman supposes (white bars in Figure D2b). The results are shown in Figure D2, compared to the data on BMI in the people of the USA [Flegal et al 2002]. In our view, none of these assumptions lead to a distribution of predictions that closely match observation, but our approach results in a distribution (Figure D2b) that has lower skew and has a lower mode than Speakman’s original analysis, and thus these new distributions are an even poorer match to the defining characteristics of the distribution of body masses in human populations as identified by Speakman [2007].

**References**

Speakman, J.R. 2007 A nonadaptive scenario explaining the genetic predisposition to obesity: the "Predation release" hypothesis. *Cell Metabol* **6**, 5-12.

Kirchengast, S. 1998 Weight status of adult !Kung San and Kavango people from Northern Namibia. *Annals Human Biol* **25**, 541-551.

Flegal, K.M., Carroll, M.D., Ogden, C.L., Johnson, C.L. 2002 Prevalence and trends in obesity among US adults, 1999-2000. *J American Med Assoc* **288**, 1723-1727.

**Figure D2:** Observed and predicted distribution in Body Mass Index given (a) individual BMI are equal to the higher threshold, (b) individual BMI are normally distributed between the two thresholds and (white bars) only the upper threshold has drifted or (black bars) both lower and upper threshold have drifted. Observed BMI in the USA as represented by Speakman (, Fig 3) shown by grey bars.


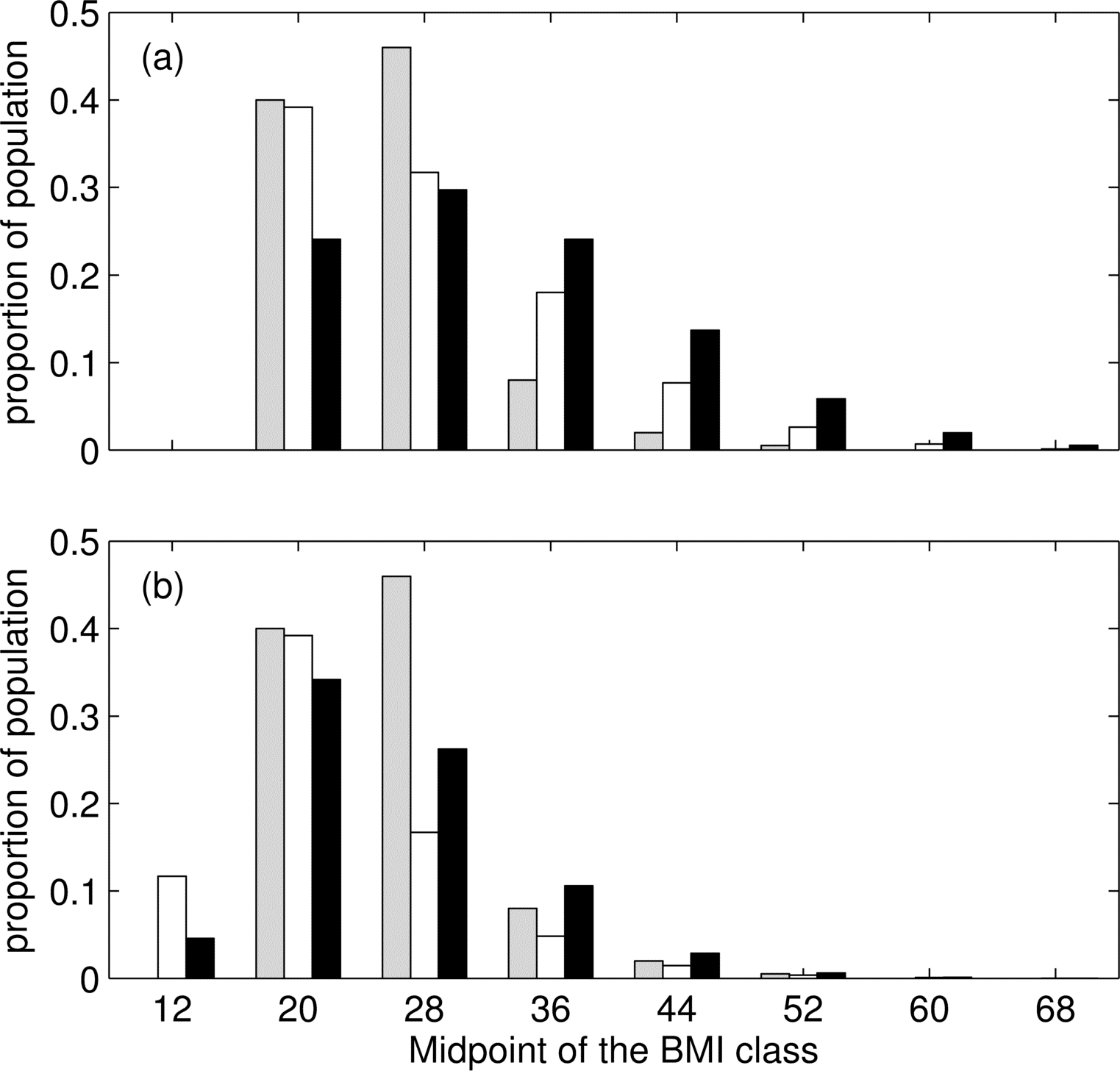

Supplement: Online Appendices [file rspb20152443supp1.doc]
